# Supplementary material for: Impact of Statin Use on Dementia Incidence in Elderly Men and Women with Ischemic Heart Disease
Source: Biomedicines. 2020 Feb 9;8(2):30. doi: 10.3390/biomedicines8020030 (PMC7168191; doi:10.3390/biomedicines8020030)
Supplement: Supplementary file 1 [file biomedicines-08-00030-s001.pdf]

Impact of Statin Use on Dementia Incidence in Elderly Men and Women with Ischemic Heart Disease

Mi–Young Kim, Minji Jung, Yoojin Noh, Sooyoung Shin, Chang Hyung Hong, Sukhyang Lee, and Yi–Sook Jung

Table S1. Subgroup analysis based on comorbidities for dementia risk according to sex.

	Dementia Incidence, n (%)														
	Total (n=71,587)					Male (n=29,012)					Female (n=42,575)				
	**n**	**case, n**	**(%)**	**HR (95% CI)**	**p**	**n**	**case, n**	**(%)**	**HR (95% CI)**	**p**	**n**	**case, n**	**(%)**	**HR (95% CI)**	**p**
Comorbidities															
Hypertension															
yes	53,541	10,937	20	0.97 (0.94-0.99)	0.021	21,464	3,507	16	0.95 (0.90-0.99)	0.039	32,077	7,430	23	0.97 (0.94-1.01)	0.107
no	18,046	3,312	18	0.85 (0.81-0.90)	<0.001	7,548	1,137	15	0.79 (0.72-0.86)	<0.001	10,498	2,175	21	0.89 (0.83-0.94)	<0.001
Diabetes															
yes	25,951	5,652	22	0.96 (0.92-0.99)	0.028	11,210	1,966	18	0.92 (0.86-0.98)	0.011	14,741	3,656	25	0.98 (0.94-1.03)	0.427
no	45,636	8,597	19	0.94 (0.91-0.97)	<0.001	17,802	2,678	15	0.92 (0.87-0.97)	0.004	27,834	5,919	21	0.95 (0.91-0.98)	0.003
Ischemic Stroke															
yes	8,935	2,606	29	1.02 (0.96-1.08)	0.550	4,334	1,100	25	0.98 (0.90-1.08)	0.739	4,601	1,506	33	1.05 (0.97-1.13)	0.211
no	62,652	11,643	19	0.93 (0.91-0.96)	<0.001	24,678	3,544	14	0.9 (0.86-0.95)	<0.001	37,974	8,099	21	0.94 (0.91-0.97)	<0.001
Depression															
yes	5,720	1,664	29	1.00 (0.93-1.07)	0.995	1,783	426	24	0.94 (0.81-1.08)	0.360	3,937	1,238	31	1.03 (0.95-1.11)	0.547
no	65,867	12,585	19	0.94 (0.91-0.96)	<0.001	27,229	4,218	15	0.92 (0.88-0.96)	<0.001	38,638	8,367	22	0.95 (0.92-0.98)	<0.001
Parkinson															
yes	712	290	41	1.02 (0.86-1.2)	0.850	263	107	41	1.18 (0.89-1.57)	0.241	449	183	41	0.94 (0.77-1.16)	0.583
no	70,875	13,959	20	0.95 (0.92-0.97)	<0.001	28,749	4,537	16	0.92 (0.88-0.95)	<0.001	42,126	9,422	22	0.96 (0.93-0.99)	0.004
Schizophrenia															
yes	137	49	36	1.31 (0.90-1.91)	0.162	62	20	32	1.04 (0.57-1.88)	0.909	75	29	39	1.52 (0.92-2.51)	0.104
no	71,450	14,200	20	0.95 (0.92-0.97)	<0.001	28,950	4,624	16	0.92 (0.88-0.96)	<0.001	42,500	9,576	23	0.96 (0.93-0.98)	0.002
